# Overcoming genetic paucity of *Camelina sativa*: possibilities for interspecific hybridization conditioned by the genus evolution pathway

**DOI:** 10.3389/fpls.2023.1259431

**Published:** 2023-09-25

**Authors:** Rostyslav Y. Blume, Ruslan Kalendar, Liang Guo, Edgar B. Cahoon, Yaroslav B. Blume

**Affiliations:** ^1^ Institute of Food Biotechnology and Genomics of National Academy of Science of Ukraine, Kyiv, Ukraine; ^2^ Institute of Biotechnology HiLIFE, University of Helsinki, Helsinki, Finland; ^3^ National Key Laboratory of Crop Genetic Improvement, Huazhong Agricultural University, Wuhan, China; ^4^ Hubei Hongshan Laboratory, Huazhong Agricultural University, Wuhan, China; ^5^ Center for Plant Science Innovation & Department of Biochemistry, University of Nebraska-Lincoln, Lincoln, NE, United States

**Keywords:** Brassicaceae, *Camelina*, evolution, false flax, polyploidy, wild relatives

## Abstract

Camelina or false flax (*Camelina sativa*) is an emerging oilseed crop and a feedstock for biofuel production. This species is believed to originate from Western Asian and Eastern European regions, where the center of diversity of the *Camelina* genus is located. Cultivated *Camelina* species arose via a series of polyploidization events, serving as bottlenecks narrowing genetic diversity of the species. The genetic paucity of *C. sativa* is foreseen as the most crucial limitation for successful breeding and improvement of this crop. A potential solution to this challenge could be gene introgression from *Camelina* wild species or from resynthesized allohexaploid *C. sativa*. However, both approaches would require a complete comprehension of the evolutionary trajectories that led to the *C. sativa* origin. Although there are some studies discussing the origin and evolution of *Camelina* hexaploid species, final conclusions have not been made yet. Here, we propose the most complete integrated evolutionary model for the *Camelina* genus based on the most recently described findings, which enables efficient improvement of *C. sativa via* the interspecific hybridization with its wild relatives. We also discuss issues of interspecific and intergeneric hybridization, aimed on improving *C. sativa* and overcoming the genetic paucity of this crop. The proposed comprehensive evolutionary model of *Camelina* species indicates that a newly described species *Camelina neglecta* has a key role in origin of tetra- and hexaploids, all of which have two *C. neglecta*-based subgenomes. Understanding of species evolution within the *Camelina* genus provides insights into further research on *C. sativa* improvements via gene introgression from wild species, and a potential resynthesis of this emerging oilseed crop.

## 1 Introduction

Recently, the *Camelina* genus has attracted attention of researchers due to one of its outstanding representatives—*С. sativa* (L.) Crantz, or false flax. This oilseed crop has emerged as a platform for a variety of genetic engineering research (Nguyen et al., 2013; Faure and Tepfer, 2016). Particularly, gene editing, aimed at modification of fatty acid composition of the seed lipids (Aznar-Moreno and Durrett, 2017; Jiang et al., 2017; Morineau et al., 2017; Ozseyhan et al., 2018; Lee et al., 2021), seed protein storage (Lyzenga et al., 2019), or production of other high-value compounds (Yuan and Li, 2020). Close relation of *C. sativa* to a model plant *Arabidopsis thaliana* (both belong to Brassicaceae Lineage I) (Warwick, 2011; Nguyen et al., 2013; Faure and Tepfer, 2016) and high amenability of the false flax to be transformed (Liu et al., 2012; Yemets et al., 2013) make this crop even more attractive for a wide range of investigations. Currently, *C. sativa* is viewed as one of the most promising feedstocks for biodiesel and aviation biofuel production (Moser, 2010; Iskandarov et al., 2014; Bansal et al., 2018; Blume et al., 2022). In addition, *C. sativa* has been described as a multipurpose crop, suitable for both food and feed purposes due to its beneficial oil composition (Ghobadi et al., 2021; Zanetti et al., 2021; Ghidoli et al., 2023). Moreover, recently, *C. sativa* was proposed as a new model crop for oilseeds, especially from the Brassicaceae family (Yuan and Li, 2020).

This domesticated species came from Europe, where this oilseed had been cultivated for a long period of time (Zubr, 1997; Vollmann and Eynck, 2015; Blume et al., 2022). In particular, *C. sativa* was one of the major oil crops in Eastern Europe until the 1940s, before it was replaced by rapeseed, sunflower, and soybean (Iljinska et al., 2007). In addition to its high amenability for genetic engineering research (Lu and Kang, 2008), false flax has received renewed attention due to its high tolerance to different abiotic factors and fungal diseases (Obour et al., 2015; Vollmann and Eynck, 2015; Berti et al., 2016), as well as for its ability to grow under unfavorable conditions (Rostami Ahmadvandi and Faghihi, 2021; Rostami Ahmadvandi et al., 2021; Ghamarnia et al., 2022; Neupane et al., 2022). However, *C. sativa* has low genetic diversity (Manca et al., 2012; Singh et al., 2015; Luo et al., 2019; Blume et al., 2020), which probably resulted from the abandonment of false flax cultivation and breeding (Brock et al., 2020; Ghidoli et al., 2023). Moreover, facultative self-pollination and self-compatibility of this crop additionally contribute to the limitation of the genetic diversity (Manca et al., 2012; Čalasan et al., 2019; Blume et al., 2020), as well as the allohexaploid nature of *C. sativa*, which originated out of at least two closely related ancestral species (Kagale et al., 2014; Mándaková et al., 2019; Zhang et al., 2020). These properties limit the improvement of this crop using both transgenic and non-transgenic approaches. Moreover, due to this fact, successful gene editing of *C. sativa* requires targeting multiple gene copies, further complicating genetic manipulation of this crop (Ghidoli et al., 2023).

Other *Camelina* species are underutilized despite their potential value as germplasm donors for *C. sativa* improvement. One candidate species for interspecific crossing may be *Camelina microcarpa*, or little-pod false flax (Brock et al., 2020), which is most likely the wild progenitor of domesticated *C. sativa* (Brock et al., 2019; Mándaková et al., 2019). This species also shows relatively high ability to form hybrids with cultivated camelina (Al-Shehbaz and Beilstein, 2010; Seguin-Swartz et al., 2013; Martin et al., 2019; Zhang and Auer, 2020). Nevertheless, other wild relatives may be used for gene introgression, but they often possess lower fertility with *C. sativa* (Seguin-Swartz et al., 2013; Zhang and Auer, 2020). Another option could be a resynthesis of this allohexaploid crop from different *Camelina* species of lower ploidy, which are closely related to the progenitors of *C. microcarpa* and cultivated *C. sativa*. Such resynthesized *C. sativa* might be used for efficient gene introgression from wild evolutionary distant *Camelina* sp., in order to boost genetic diversity of this crop and increase breeding programs efficiency. This has already been successfully done for synthetic hexaploid wheat (Li et al., 2018) and allotetraploid *Brassica napus* crop (Becker et al., 1995; Girke et al., 2012). At the moment, breeding of *C. sativa* is restricted by low number of available cultivars, most of which are suffering from low seed yields (Zanetti et al., 2021; Ghidoli et al., 2023). The use of such a synthetic hexaploid may be almost the only approach (with the exception of transgene-based methods) that can potentially help to solve the problems mentioned. However, creation of “synthetic hexaploid camelina” requires the best comprehension of origin and evolution of this species.

The history of the *Camelina* genus is complex and includes several evolutionary events that led to the formation of complex allopolyploid species, which is common to most flowering plant genomes (Wang et al., 2019). The hexaploid genome of *C. sativa* consists of three subgenomes, two of which appeared to be very closely related (Kagale et al., 2014). With the description of a new diploid species—*Сamelina neglecta* (Brock et al., 2019)—the understanding of the *Camelina* genus evolution has significantly improved, based on the comparative data of fluorescence *in situ* hybridization and reconstruction of ACK block allocation with the genome of *C. sativa* (Mándaková et al., 2019). Subgenomes of *C. sativa* did not undergo chromosomal rearrangements after polyploidization (Mándaková et al., 2019), like other meso- or neopolyploid species such as *B. napus* (Zhang et al., 2018; Chawla et al., 2021). The collinearity of major wild relative genomes with *C. sativa* subgenomes was revealed later (Chaudhary et al., 2020), together with major evolutionary trajectories and karyotype changes that were identified or confirmed (Zhang et al., 2020). Phylogenetic relationships between representatives of the genus were also studied, but their results differed, depending on the chosen method and the investigated region of *Camelina* distribution (Brock et al., 2018; Čalasan et al., 2019; Brock et al., 2022a; Brock et al., 2022b). However, not all aspects of *Camelina* genus evolution and hexaploid species divergence have been investigated yet. Understanding the evolutionary trajectories that led to the origin of *C. sativa* is even more interesting, since this crop has been proposed as the model species for the study of plant polyploidy (Mándaková et al., 2019). At the same time efficient gene introgression via interspecific hybridization requires a comprehensive understanding of the genome evolution of this complex allohexaploid.

Therefore, in this paper, we propose and discuss an integrated model of the evolution of the genus *Camelina*, the origin of polyploid species, divergence, and domestication of hexaploids, based on recent research findings. The proposed model covers the entire history of *Camelina* genus evolution, describes the main events of species origin, and takes into account a wide range of data on genomics, cytogenetics, population genetics, phylogenetics, and interspecific hybridization research. A proposed view of the origin of *C. sativa* enables further improvement of this emerging oilseed via introgression of new allelic variants or even traits from wild hexaploid relatives or from resynthesis of allohexaploid camelina from its tetra- and diploid relatives.

## 2 Evolution of *Camelina* species

### 2.1 Understanding of the origin of hexaploid *Camelina* species

In recent years, the origin of the hexaploid species *Camelina*, *C. sativa*, has been of great interest as this species is defined as an extremely attractive plant platform for genetic engineering research, including research related to lipid metabolism (Nguyen et al., 2013; Faure and Tepfer, 2016; Ghidoli et al., 2023). In addition, the fact that *C. sativa* is the only allohexaploid in the tribe Camelineae and that this species is a close relative of *A. thaliana* (both are Brassicaceae Lineage I representatives) makes *C. sativa* a very attractive model for studying plant polyploidy. Previously, the view of the origin of *C. sativa* was largely limited to the understanding that this species originated in southeastern Europe and southwestern Asia (Radatz and Hondelmann, 1981), most likely in the regions adjacent to the Black Sea.

The first attempt to understand the structure of the genome and thus to suggest the evolutionary path of *C. sativa* was made by Gehringer et al. (2006). These authors noted for the first time that amplification of linked SSR markers linked to fatty acid desaturase gene in *C. sativa* results in the amplification of multiple alleles, suggesting the polyploid nature of this crop. Next steps were made by Hutcheon et al. (2010), who studied *FAD2* and *FAE1* genes of *Camelina* species. The authors found that *C. sativa* and *C. microcarpa* were potentially hexaploid species, whereas *Camelina rumelica* was tetraploid. The authors also found that *C. sativa* and *C. microcarpa* have two out of the three almost identical copies of genes of common origin, whereas the origin of the third copy was intended for different species with lower ploidy. In addition, the polyploid status of *C. sativa* was confirmed by Galasso et al. (2011) by comparing the number of β-tubulin gene orthologs in *C. sativa* and in *A. thaliana*.

The next major advance in understanding the evolution of *Camelina* came after the whole-genome sequencing and assembly of the *C. sativa* genome by Kagale et al. (2014), who confirmed the hexaploid status of this species. Three subgenomes were identified in the *C. sativa* genome, which had a highly undifferentiated structure and significantly rearranged chromosomes in each of the subgenomes, compared with the ancestral cruciferous karyotype (ACK) and the diploid ancestral karyotype of *Camelina* (CAM) (Kagale et al., 2014; Mándaková et al., 2019). Additionally, the authors found a phylogenetic relationship between *C. sativa* subgenomes and lower-ploidy species. The *C. sativa* subgenome 3 (CsG-3) was found to have higher collinearity with *Camelina hispida*, whereas the other two subgenomes were more similar to different *C. rumelica* accessions (Kagale et al., 2014). Later, Galasso et al. (2015) identified a specimen of *C. microcarpa* with significantly reduced ploidy (2*n*=12, USDA-NPGS accession ID: PI 650135) and a distinct profile of amplified β-tubulin-based markers. Taking into account the results of molecular analysis and the combination of morphological features, a new diploid *C. neglecta* was described based on this specimen (Brock et al., 2019), which allowed to include this new species in the genome analysis of representatives of the genus *Camelina.*

Mándaková et al. (2019) proposed for the first time a detailed model of genome evolution of *Camelina* species based on the data of genomic hybridization *in situ* and other methods of comparative genomics. In terms of the proposed model, it was found that *C. neglecta* contributed to the formation of CsG-1 and Cs-G2, whereas Cs-G3 is more collinear with *C. hispida*, as previously shown by Kagale et al. (2014). This evolutionary scheme indicates that the karyotype of *C. neglecta* (*n* = 6) originated from the ancestral *C. neglecta*-like karyotype (*n* = 7). The reduction in the number of chromosomes in modern *C. neglecta* was achieved by the association (end-to-end translocation) of chromosomes CAM6 and CAM7 (CAM—the ancestral *Camelina* karyotype), which led to the origin of the Cn6 chromosome of *C. neglecta*, which is the largest chromosome in *C. sativa* genome—Cs11 (Cs-G1) (**Table 1**). Moreover, *C. rumelica* was found to be an allotetraploid, resulting from hybridization of *C. neglecta* and *C. hispida* (Mándaková et al., 2019), which may explain the previously observed by Kagale et al. (2014) high karyotype collinearity between *C. rumelica* and the third subgenome of *C. sativa*. It should be noted that for the majority of *Camelina* polyploids, no signs of translocations between subgenomes were found, which indicates a high stability of the karyotype after interspecific hybridization (Mándaková et al., 2019).

**Table 1:** Subgenomes of *C. sativa* and their possible progenitors, according to the recent data.

Subgenome 1 (N, or N^6^ genome) A genome	Subgenome 2 (N, or N^7^ genome) B genome	Subgenome 3 (H, or H^7^ genome) C genome
Csa04, Csa07, Csa08, Csa11, Csa14, Csa19	Csa01, Csa03, Csa06, Csa10, Csa13, Csa16, Csa18	Csa02, Csa05, Csa09, Csa12, Csa15, Csa17, Csa20
*C. neglecta* (*n* = 6)	Supposed *C. neglecta*-like ancestor (*n* = 7)*	*C. hispida* subsp. *hispida* (*n* = 7)
Cn1, Cn2, Cn3, Cn4, Cn5, Cn6	CAM1, CAM2, CAM3, CAM4, CAM5, CAM6, CAM7	Ch1, Ch2, Ch3, Ch4, Ch5, Ch6, Ch7

* - karyotype is highly similar to CAM.

These findings were later confirmed by Chaudhary et al. (2020), who performed genotyping by sequencing of 193 accessions of different representatives of the genus *Camelina*. The authors found that the subgenomes of tetraploid cytotype of *C. microcarpa* could only be related to CsG-1 and CsG-2 of *C. sativa*, which are believed to be the result of hybridization between *C. neglecta* and ancient *C. neglecta*-like karyotypes, originally described by Mándaková et al. (2019). Furthermore, the origin of *C. rumelica* was confirmed by Chaudhary et al. (2020), but the authors noted that its subgenomes did not share the genomic pattern with CsG-1 and CsG-3 of *C. sativa*, indicating that this species might not be a direct ancestor of *C. sativa*, which was consistent with previous findings of Mándaková et al. (2019). The karyotype evolution of *Camelina* species, reported by Mándaková et al. (2019), was also confirmed by Zhang et al. (2020), in which the distribution of ACK genomic blocks in *C. sativa* chromosomes was reconstructed *de novo* using *A. lyrata* as internal reference. The relatively recent genome sequencing of diploid *Camelina* species (*Camelina laxa*, *C. neglecta*, *C. hispida* var. *hispida*, and var. *grandiflora*) confirmed previous findings on the evolution of *Camelina* genomes and also highlighted the crucial role of these species in the formation of polyploid taxa within the genus (Martin et al., 2022; Chaudhary et al., 2023). Research of Čalasan et al. (2019) and Brock et al. (2020) greatly contributed to understanding of the *C. microcarpa* distribution and cytotype/ribotype composition of this taxon (hexaploid *C. microcarpa* is represented by 2*n* = 40 and 2*n* =38 cytotypes, types 1 and 2, respectively). A study by Brock et al. (2022a), (2022b) shed light on cytoplasmic inheritance within the *Camelina* genus, showed the executive role of *C. neglecta* in the origin of polyploid taxa, and confirmed the close relationship of *C. microcarpa* (2*n* = 40) with domesticated *C. sativa* (2*n* = 40). Finally, a recent study by Mandáková and Lysak (2022) revealed the nature of the “missing link” in *Camelina* genus—the tetraploid *Camelina intermedia* nom. prov. (2*n* = 26, formerly known as *C. microcarpa* tetraploid or *C. neglecta*-like tetraploid).

Dating the evolutionary events that took place within the genus *Camelina* turned out to be a non-trivial task. First attempts to estimate divergence time of *Camelina* species were made using 18S ETS sequences to reconstruct phylogeny and date evolutionary events (Čalasan et al., 2019). However, this approach faced several obstacles. In particular, sequencing of a single copy of the ETS from polyploid *Camelina* species may result in extraction of sequences from a specific subgenome, which may affect the topology of the inferred tree. Čalasan et al. (2019) reported *C. microcarpa* as a polyphyletic group consisting of several ribotypes, which may, however, be influenced by sequence samples isolated from highly similar N^6^ and N^7^ subgenomes. Similarly, *C. hispida* was placed together with *C. sativa*, which was most likely due to extraction of ETS sequences corresponding to the H^7^ subgenome. In addition, Kwiatek et al. (2021) later reported that the number of different rDNA loci may vary within different genotypes of *C. sativa* and *C. microcarpa*, which may further complicate such studies. However, to date, Čalasan et al. (2019) reported the most comprehensive study of nuclear marker-based divergence dating of *Camelina* species. Thus, their result should be taken into account but with the caveat that the datings rather represent the divergence time of (sub)genomes, especially for polyploid taxa.

Another recent study by Brock et al. (2022a) attempted to date evolutionary events within the *Camelina* genus, based on chloroplast genome phylogeny. The authors used a wider panel of species and achieved dates accurate enough to distinguish cytoplasmic lineages. The majority of the evolutionary events were dated without “subgenome bias”; however, some aspects still remain unresolved. For example, the phylogeny reconstruction, proposed by Brock et al. (2022a) identifies *C. rumelica* as a polyphyletic group in relation to *C. hispida*. Similarly, *C. microcarpa* cytotypes and *C. sativa* often shared common clades. In view of the described questions, the results of both studies by Čalasan et al. (2019) and Brock et al. (2022a) were taken into account in the evolutionary model, described below.

### 2.2 Current model of *Camelina* evolution: early *Camelina* divergence and origin of diploids

**Figure 1:**
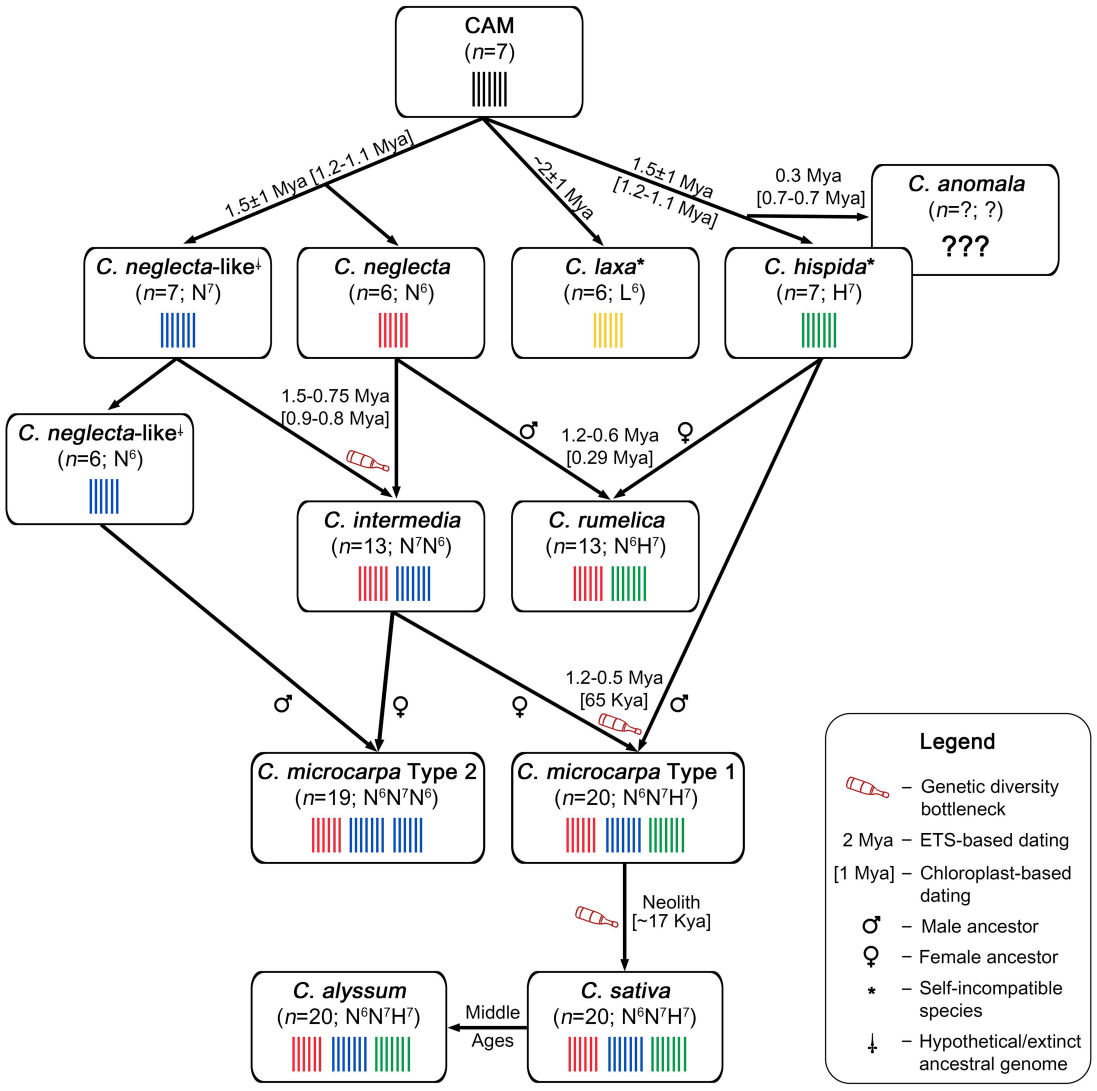
Integrated model of *Camelina* species evolution, based on the recent studies.

Based on the recently described findings, here we propose an integrated evolutionary model for the *Camelina* genus (**Figure 1**). The proposed model relies on the most recent research on *Camelina* genetics, genomics, and phylogenetics (Kagale et al., 2014; Kim et al., 2017; Čalasan et al., 2019; Mándaková et al., 2019; Brock et al., 2020; Chaudhary et al., 2020; Zhang et al., 2020; Chaudhary, 2021; Brock et al., 2022a; Brock et al., 2022b; Martin et al., 2022; Chaudhary et al., 2023).

It has been suggested that the divergence of the ancestral *Camelina* karyotype (the last common ancestor for the genus) occurred approximately 2.5 ± 1 Mya (Čalasan et al., 2019; Brock et al., 2022a) as the time of *Camelina* speciation among other Camelineae representatives. The next major evolutionary event, the divergence of *C. laxa* species from the other *Camelina* lineage, occurred approximately 2 ± 1 Mya, according to the ETS-based dating (Čalasan et al., 2019). Chloroplast-based dating suggests that the diversification of *C. laxa* cytoplasmic lineage took place approx. 1.62 Mya (estimated dating for most recent common ancestor of *Camelina* spp.) (Brock et al., 2022a) (**Figure 1**). The basal status of *C. laxa* and its significant genetic differences from other *Camelina* sp. have also been confirmed by numerous studies (Hutcheon et al., 2010; Kagale et al., 2014; Brock et al., 2018; Mándaková et al., 2019; Chaudhary et al., 2020; Martin et al., 2022).

Most studies show that *C. hispida* was the next species to diverge from the genus along with others (Hutcheon et al., 2010; Kagale et al., 2014; Brock et al., 2018; Mándaková et al., 2019; Brock et al., 2022a; Martin et al., 2022). Instead, results of Čalasan et al. (2019) showed that *C. hispida* belongs to the same clade as *C. sativa* and *Camelina alyssum*, which is most likely caused by the above-described “subgenomic bias” of the analyzed ETS sequences. Thus, we suggest that the diversification event of *C. hispida* (**Figure 1**) occurred approximately 1.5 ± 1 Mya, if dated by the divergence of ETS sequences (Čalasan et al., 2019). Chloroplast-based dating also suggests that speciation of *C. hispida* occurred 1.2–1.1 Mya (Brock et al., 2022a). It is also worth noting that *C. hispida* has highly shattered chromosomes, if compared with CAM (Mándaková et al., 2019; Martin et al., 2022). Furthermore, this species is the only *Camelina* diploid that has infraspecific taxa, namely, var. *hispida*, var. *grandiflora*, var. *lasiocarpa*, and var. *steifelhagenii*.

A recent study of Martin et al. (2022) revealed genomic differences between *C. hispida* var. *hispida* and var. *grandiflora*. Divergences between these infraspecies may be a potential explanation for the polyphyly of *C. hispida*, observed by Brock et al. (2022a) in their study. The ploidy and phylogeny of the other two infraspecies (var. *lasiocarpa* and var. *steifelhagenii*) remain a mystery; therefore, the divergences between these varieties are not shown in **Figure 1**. It should be noted that *C. laxa* and *C. hispida* are the only self-incompatible species of the genus *Camelina* (the status of *Camelina anomala* is currently unknown) (Čalasan et al., 2019). At the same time, another diploid taxon, *C. neglecta*, is a facultatively self-pollinated plant, similar to *C. sativa*, *C. alyssum*, and *C. microcarpa*.

The evolutionary pathway of *C. anomala* cannot currently be adequately described due to the lack of experimental data. This species was not found for a long time because of its very limited geographical distribution (Brock et al., 2018). Consequently, the lack of fresh plant material or seeds of *C. anomala* significantly limits its use in genomic studies. The chromosome number and ploidy status of this species are currently unknown. Previously, *C. anomala* was discussed as an ancestral species for the whole *Camelina* genus due to its atypical morphology (Mirek, 1984). On the contrary, the results of Čalasan et al. (2019) suggest that *C. anomala* may be the closest relative of *C. hispida*, which diversified probably less than 0.3 Mya, whereas the data of Brock et al. (2022a) suggest that *C. anomala* is basal species for *C. hispida*–*C. rumelica* lineage and its speciation event occurred much earlier—approx. 0.7–0.5 Mya (**Figure 1**). *C. anomala* has a robust habitus, particularly elongated silicles (alike *Brassica* sp.) (Mirek, 1984), which makes this species a very attractive target for research, given the possibility of introducing these traits into the cultivated *C. sativa* to promote its seed productivity and overall yields. However, due to the unknown status of ploidy, it is not yet possible to draw a definitive conclusion on the possibility of interspecies hybridization of *C. anomala*.

As shown in **Figure 1**, according to chloroplast-based datings, the lineages of *C. neglecta* and *C. hispida* diverged approximately 1.2–1.1 million years ago (Brock et al., 2022a). Unfortunately, Čalasan et al. (2019) did not include *C. neglecta* in their ETS-based phylogeny reconstruction, so no nuclear marker-based dating is available for this species. The ancestor of *C. neglecta* is thought to have contained seven chromosomes (*C. neglecta*-like, *n* = 7, N^7^). To date, no living populations of the *C. neglecta*-like (*n* = 7, N^7^) ancestor have been found in nature (Mándaková et al., 2019; Brock et al., 2022a; Mandáková and Lysak, 2022).

Unlike other *Camelina* diploids, the date of origin of *C. neglecta* from its presumed *C. neglecta*-like (N^7^) ancestor (Mándaková et al., 2019) is currently unknown (**Figure 1**). *C. neglecta* (*n* = 6; N^6^) is thought to have descended from its ancestor of a higher chromosome number as a result of descending dysploidy (Mandáková and Lysak, 2018), sometimes called “mixoploidy” in Brassicaceae species (Kunakh et al., 2008). Signs of this evolutionary event are evident in the presence of the so-called fusion chromosome (Cn6, homolog in *C. sativa*—Cs11), which is one of the largest chromosomes among all *Camelina* sp. karyotypes (Mándaková et al., 2019; Chaudhary et al., 2020; Zhang et al., 2020). It is noteworthy that *C. neglecta* is characterized by the smallest genome size within the genus (Chaudhary et al., 2020; Martin et al., 2022; Chaudhary et al., 2023).

As reported by Brock et al. (2019), *C. neglecta* is currently found in one location, so only little is known about its distribution and wild population structure. Potentially, reported findings of *n* = 6 C*. rumelica* in Hungary and the USA and 2*n* = 12 C*. sativa* (Warwick and Al-Shehbaz, 2006) may correspond to unidentified specimens of *C. neglecta* (Brock et al., 2019), which needs to be confirmed. It was later supposed that *C. neglecta* may be distributed throughout the Eurasian steppe (Brock et al., 2022a). In addition, when collecting *Camelina* sp. in Turkey, Brock et al. (2018) identified a potentially diploid accession (JRB30 in original paper), which had a genome size of 370 Mbp, which is very close to *C. laxa* and *C. hispida*. However, this species is grouped in the *C. rumelica* clade, as a basal branch, apart from the diploid *Camelina* clades, so potentially this accession may correspond to a *C. neglecta*-like species (morphological data did not support this) or another unidentified diploid species, probably, close to *C. hispida* (**Figure 1**).

### 2.3 *C. neglecta* plays a key role in the origin of *Camelina* polyploids

#### 2.3.1 Origin of *Camelina* tetraploid species

The genus *Camelina* is currently believed to include at least two distinct tetraploid species, formation of which has been mediated by *C. neglecta* (Mándaková et al., 2019). The first of them is the tetraploid cytotype of *C. microcarpa* (*n* = 13), which was also called tetraploid *C. neglecta*-like by Brock et al. (2022a) and was recently recognized by Mandáková and Lysak (2022) as a potentially separate species—*C. intermedia* nom. prov.

According to Mándaková et al. (2019), it was supposed that evolutionary transition of *C. neglecta*-type subgenomes N^6^ and N^7^ to CsG-1 and Cs-G2 of *C. sativa* was mediated by this tetraploid cytotype of *C. microcarpa*, which was at that time theoretically defined as the “tetraploid *C. neglecta*-like” ancestor with *n* = 13 karyotypes. Recent findings of Chaudhary et al. (2020) have revealed that the tetraploid *C. microcarpa* (*n* = 13) cytotype (collected in Canada) carries both *C. neglecta*-type subgenomes (N^6^ and N^7^) and shares their structure with hexaploid *C. microcarpa* and *C. sativa* (**Figure 1**). This makes the tetraploid *C. microcarpa* an ideal candidate for this “intermediate tetraploid,” as suggested by Mándaková et al. (2019). A study by Brock et al. (2022a) confirmed the close relationship of this cytotype with the maternal lineage of *C. neglecta* and showed its intermediate position between *Camelina* diploids and hexaploids. Recently, Mandáková and Lysak (2022) found that representatives of this tetraploid cytotype (which was proposed to be called *C. intermedia*) are widespread in the Eurasian Steppe belt, in particular in Kazakhstan, Mongolia, and South-Eastern Russia, where corresponding specimens of this cytotype had been previously collected by Čalasan et al. (2019).

It is believed that *C. intermedia* (≡tetraploid cytotype of *C. microcarpa*) appeared as a result of hybridization between *C. neglecta* (*n* = 6, N^6^) and its ancestral form—*C. neglecta*-like (*n* = 7, N^7^). Most likely, after the speciation of *C. neglecta* (reduction in the number of chromosomes), two populations of such closely related species of N^6^ and N^7^ genomes hybridized, which led to the formation of tetraploid cytotypes *C. microcarpa* and *C. intermedia* (2*n* = 26; N^6^N^7^) (**Figure 1**). According to the results of Čalasan et al. (2019), this event could have occurred between 1.5 and 0.75 million years ago (divergence between the *C. microcarpa*–*C. rumelica* clade and the *C. microcarpa* clade, which included *C. intermedia* specimens). The results of chloroplast-based dating are consistent with the described findings and suggest that *C. intermedia* arose approximately 0.9–0.8 Mya (Brock et al., 2022a).

Mándaková et al. (2019) and Brock et al. (2022a) described this interspecific hybridization event as “auto-alloploidy,” to underline the extremely close relation between N^7^ and N^6^ genomes. However, some authors referred that the origin of the tetraploid *C. microcarpa* (*C. intermedia*) could be the result of the autopolyploidy (Kagale et al., 2014; Faure and Tepfer, 2016; Hotton et al., 2020). However, comparative genomics results do not support WGD-mediated origin of *C. intermedia*. Both Zhang et al. (2020) and Mandáková and Lysak (2022) in their analyses of *Camelina* karyotype evolution noted that the different allocation of ACK blocks in the chromosomes of N^6^ and N^7^ genomes serves an evidence for the separate evolution of *C. neglecta* and *C. neglecta-like* lineages, since N^6^ and N^7^ genomes each have unique translocations, for instance, presence of a fused chromosome in N^6^ subgenomes of polyploid *Camelina* sp. and the parental *C. neglecta* and reduced size of the *C. neglecta* genome (Zhang et al., 2020; Mandáková and Lysak, 2022; Martin et al., 2022; Chaudhary et al., 2023). Furthermore, it is highly unlikely that the fusion of the *C. neglecta* chromosome (Cn6) was split again into two separate chromosomes in N^7^, which are identical to the ancestral CAM chromosomes. Therefore, it should be assumed that *C. intermedia* inherited N^6^ and N^7^ (sub)genomes from separate diploid species, one of which (*n* = 7) is currently unidentified or completely extinct.

Another tetraploid species, *C. rumelica* (*n* = 13, N^6^H^7^), most likely arose as a result of hybridization between *C. neglecta* (N^6^) and *C. hispida* (H^7^) (Mándaková et al., 2019; Chaudhary et al., 2020), which is shown in **Figure 1**. This species is the second most widespread wild *Camelina* species after *C. microcarpa*, whereas the distribution of the majority of wild taxa of the genus is limited to the center of their origin, the Irano-Turanian Region (e.g., *C. laxa*, *C. hispida*) (Eliáš et al., 2014; Čalasan et al., 2019). The origin of this species was dated to 1.2–0.6 Mya using ETS sequences and to 0.29 Mya using a phylogeny reconstruction based on the chloroplast genome (Čalasan et al., 2019; Brock et al., 2022a). Notably, *C. rumelica* is the only polyploid *Camelina* species that inherited maternal cytoplasm from *C. hispida* (**Figure 1**) (Brock et al., 2022a). The species is represented by separate subspecies (subsp. *rumelica* and subsp. *transcaspica*); however, no data on their genome or genetic differences have yet been reported.

It should be noted that *C. rumelica* is almost the only species within the genus that faced post-polyploidization changes in the genome structure. While there was no recombination between the subgenomes, both N^6^ and H^7^ genome components showed signs of chromosome rearrangements. Mándaková et al. (2019) indicated that *C. rumelica* carries signs of one post-allopolyploidization translocation in the H^7^ subgenome component whereas the N^6^ subgenome underwent a complete rearrangement. Later, Chaudhary et al. (2020) found that both N^6^ and H^7^ subgenomes of modern *C. rumelica* do not share the same subgenome structure with *C. sativa* and, therefore, does not have the same chromosomal organization as parental species, such as *C. hispida*. Both mentioned studies included only a limited number of specimens *C. rumelica* (as well as only a few specimens of *C. hispida* and a single specimen of *C. neglecta*). Given the fact that both *C. hispida* and *C. rumelica* have infraspecies with potentially different genome structures, it may be assumed that these subspecies/varieties could have different evolutionary relationships. Even more, *C. rumelica* subsp. *rumelica* is a self-compatible plant, whereas subsp. *transcaspica* appears to be the only self-incompatible *Camelina* polyploid (Seguin-Swartz et al., 2013).

Additionally, Brock et al. (2022a) reported about the polyphyly status of *C. hispida* and *C. rumelica* clades, which serves as an additional evidence for the distinct evolutionary relationship of the *C. rumelica* subspecies with *C. hispida* infrataxa. Unfortunately, the intraspecific taxa of these species are rarely considered separately in phylogeny studies, so little is known about their genetics and evolution.

#### 2.3.2 Possible pathways of hexaploid *Camelina* species origin

To date, it has been reported that three hexaploid species can be found in the *Camelina* genus. Among them, there are two cultivated species, *C. sativa* and *C. alyssum*, which are reported to have 2*n* = 6x = 40 chromosomes; also, *C. microcarpa* wild species aggregate, consisting of two distinct cytotypes with different numbers of chromosomes: type 1 (2*n* = 6x = 40) and type 2 (2*n* = 6x = 38) (Warwick and Al-Shehbaz, 2006; Mándaková et al., 2019; Brock et al., 2020; Chaudhary et al., 2020; Brock et al., 2022a; Brock et al., 2022b; Mandáková and Lysak, 2022).

The consensus view on the origin of *C. microcarpa* type 1 (*n* = 20) suggests that this cytotype could arise as a result of a possible relationship between the *C. intermedia* (tetraploid *C. microcarpa* form (*n* = 13, N^6^N^7^) and *C. hispida* (*n* = 7, H^7^), which is shown in **Figure 1** (Mándaková et al., 2019; Chaudhary et al., 2020; Mandáková and Lysak, 2022). The event of hybridization of *C. intermedia* and *C. hispida* can be dated to 1.2–0.5 Mya, based on the ETS sequence analysis, reported by Čalasan et al. (2019), if the divergence within the *C. microcarpa*–*C. rumelica* clade is considered. However, since the topology of the reported ETS-based tree is unlikely to be consistent with the recent genomics data, the chloroplast-based datings of Brock et al. (2022a) may be considered as more accurate, suggesting that this event occurred approximately 65 Kya (**Figure 1**). It should be noted that *C. microcarpa* type 1 inherited two of the three subgenomes from *C. neglecta*-like species (Chaudhary et al., 2020; Mandáková and Lysak, 2022). Recent data indicate that *C. intermedia* served as a maternal plant during interspecific hybridization with *C. hispida* (**Figure 1**). Therefore, *C. microcarpa* type 1 also belongs to the maternal lineage of *C. neglecta* (Brock et al., 2022a).

*C. microcarpa* type 1 is believed to be a direct progenitor of cultivated *C. sativa*. Recent findings suggest that the domestication event might have occurred ~17 kya, which roughly corresponds to the origin of agriculture (Brock et al., 2022a; Brock et al., 2022b). Archaeological findings support these data (domestication was dated to 9–12 kya) and point out that Caucasus may be the center of domestication of *C. sativa* (Hovsepyan and Willcox, 2008). This theory is supported by the fact that the distribution of *C. microcarpa* type 1 might be limited to the Caucasian region (Brock et al., 2020; Chaudhary et al., 2020), whereas the Caucasus itself is included into the center of *Camelina* genus diversity (Čalasan et al., 2019). The genome structure and chromosome number of *C. sativa* are similar to those of *C. microcarpa* type 1 (2*n* = 6x = 40, N^6^N^7^H^7^) (Mándaková et al., 2019; Chaudhary et al., 2020), and this species also shares a common *C. neglecta*-type maternal lineage with its progenitor (Brock et al., 2022a; Martin et al., 2022).

Another hexaploid cultivated species, *C. alyssum*, has the same genome structure as *C. sativa* and is considered to be a descendant of the latter (Chaudhary et al., 2020). There are no reliable molecular dating for this species. Čalasan et al. (2019) reported that *C. alyssum* may diverge between 0.3 and 0.2 million years ago; however, this dating had very modest statistical support. At the same time, no chloroplast genome-based dating was reported for this species. The earliest reported archeological finds of *C. alyssum* were dated to no earlier than the Middle Ages (Kroll, 1999; Woch, 2012).

It is very likely that *C. alyssum* originated from *C. sativa* in flax fields (**Figure 1**), as a result of mimicry-targeted selection, which induced changes in the fruit morphology, plant habitat, and type of fruit-ripening to resemble such traits of flax (Barrett, 1983; Čalasan et al., 2019). Genetically determined differences between *C. alyssum* and *C. sativa*, associated with the flax mimicry, were also identified quite a long time ago (Zinger, 1909). *C. alyssum* is also known to share the same distribution dynamics with flax (Stebbins, 1950; Vavilov, 1992). Currently, *C. alyssum* is almost extinct from Central Europe (Francis and Warwick, 2009). Absence of this species in gene banks (and potentially its misidentification with *C. sativa*) raised debates about the status of this taxa. However, this species (and its potential hybrids with *C. sativa*) can still be found in Eastern Europe, particularly in Ukraine (Iljinska et al., 2007). This species has been reported to have pollen and seed morphology distinct from *C. sativa* (Bojnanský and Fargašová, 2007; Sagun and Auer, 2017). In addition, *C. alyssum* has increased seed size, compared with *C. sativa* (Mirek, 1980). This trait may be of interest for its introgression into cultivated *C. sativa* lines, in order to improve seed performance and yield.

It is sometimes discussed that the ancestral or modern form of *C. rumelica* may have served as an intermediate donor of subgenome components H^7^ and N^6^ during the origin of the *C. sativa*–*C. microcarpa* type 1 hexaploid lineage (Dorofeyev, 2019; Hotton et al., 2020). Such a contradictory point of view is also partially supported by the data of Čalasan et al. (2019), who reported that *C. rumelica* and *C. microcarpa* belong to a common clade, separate from the *C. sativa*–*C. alyssum* lineage. However, the chances of successful formation of *C. microcarpa* type 1 (N^6^N^7^H^7^) via the hybridization between *C. rumelica* (N^6^H^7^) and *C. intermedia* (N^6^N^7^) are quite low. In the case of such hybridization between tetraploid species sharing one of the two subgenomes, the chances of stable allohexaploid hybrids are very low.

The impossibility of such crosses was shown by the example of interspecific crosses between *Brassica* allotetraploids (amphidiploids), which have the same subgenomes, for example between *B. napus* (AACC) and *Brassica juncea* (AABB). The most frequent result of such interspecific crosses is the formation of AABC tetraploids, in which B–C components encounter allosynthetic associations in meiosis and produce B–C genome association (Mason et al., 2010). The chances of obtaining allohexaploids from such a crossing of two closely related tetraploids are very low (Katche et al., 2021). More successfully, hexaploids can be formed by hybridizing a tetraploid with a diploid, which was shown in the example of *Brassica* species (Gaebelein et al., 2019) and in a classical example of soft wheat (Li et al., 2018). Finally, *C. rumelica* has been reported to have a different organization of N^6^ subgenome component compared with *C. intermedia* or to hexaploids *Camelina* sp. (Mándaková et al., 2019; Chaudhary et al., 2020; Mandáková and Lysak, 2022). Given the above, it is highly likely that *C. microcarpa* type 1 and its descendants arose through interspecific hybridization between *C. intermedia* and *C. hispida*.

The last among the mentioned *Camelina* allohexaploids is the recently described *C. microcarpa* type 2 (*n* = 19). The origin of this cytotype is still a mystery. Chaudhary et al. (2020) reported that *C. microcarpa* type 2 shares two subgenomes with *C. microcarpa* type 1, particularly the first and second subgenomes (N^6^ and N^7^), which were most likely inherited from a tetraploid currently named *C. intermedia*. The origin of the third subgenome of this cytotype is largely unknown. This subgenome has been reported to show signs of reduced chromosome number and also to have a “fusion chromosome” (Chaudhary et al., 2020). Finally, it was indicated that the third genome of *C. microcarpa* type 2 is more homologous to the *C. neglecta*-type genome, than to the *C. hispida* genome (Chaudhary et al., 2020). Later, it was reported that the third genome of *C. microcarpa* type 2 is likely to originate from a 6-chromosomal *C. neglecta*-like ancestor, which diverged more recently, than *C. neglecta* itself (Chaudhary, 2021), giving rise to this cytotype. The only taxon containing three *C. neglecta*-like subgenomes is N^6^N^7^N^6^. Such view is largely supported by Mandáková and Lysak (2022), as Brock et al. (2022a) reported that both cytotypes of hexaploid *C. microcarpa* are likely to have a common tetraploid maternal species (*C. intermedia*) but different paternal diploid progenitors. However, this question requires further research, as this *C. microcarpa* cytotype appears to be more widespread, than its type 1 relative (Brock et al., 2022b). The described consensus view on the origin of *C. microcarpa* type 2 is shown in **Figure 1**.

Considering the above, it can be concluded that *C. neglecta*-like genomes have played a crucial role in the evolution of *Camelina* polyploids, as each of them contains at least one N-type subgenome (**Figure 1**). Even more, *C. microcarpa* type 1, cultivated *C. sativa*, and *C. alyssum* have inherited two N-type subgenomes, whereas their relative, *C. microcarpa* type 2, could carry three *C. neglecta*-type genomes. Finally, all hexaploids and tetraploids *C. intermedia* belong to the *C. neglecta* maternal lineage. Such exceptional role of *C. neglecta* in the genus evolution makes it an ideal candidate for a model species, which can be used as a platform for biotechnological and genomic research.

### 2.4 Subspecies of *C. hispida* had different roles in origin of hexaploid *Camelina*

As noted above, *C. hispida* is known to have several infraspecies, including var. *hispida* and var. *grandiflora*. As in the case of *C. rumelica*, the status and recognition of these varieties are still a matter of debate. However, it has been recently shown that these varieties differ in genome size: smaller in *C. hispida* var. *grandiflora*—0.65 ± 0.03 pg—and greater in *C. hispida* var. *hispida*—0.73 ± 0.02 pg (Wu, 2016). This opens a valid reason for further revision of the taxonomic structure within the *C. hispida*, as well as to identify which of the known *C. hispida* varieties is closer to the H^7^ subgenomic component of *C. sativa* (**Figure 1**).

For instance, Chaudhary et al. (2020) investigated only *C. hispida* var. *grandiflora* (given the taxonomy of the accession, used in the mentioned study), which could potentially have a smaller genome size (0.59 ± 0.02 pg in this study), and found that its genome structure is not as highly collinear with Cs-G3, as previously reported by Mándaková et al. (2019) for *C. hispida*. On the contrary, Mándaková et al. (2019) and Brock et al. (2020) most likely used samples of *C. hispida* var. *hispida* in their genomic studies (taking into account the taxa recognition, made by the authors) collected in the center of origin (Turkey). Thus, there is a very high probability that *C. hispida* var. *hispida* is the exact progenitor of Cs-G3, but not the *C. hispida* var. *grandiflora*.

Recent results by Martin et al. (2022) strongly support these assumptions. It was demonstrated that *C. hispida* var. *hispida* maintains a more similar genome structure to the third (H^7^) subgenome of *C. sativa* than *C. hispida* var. *grandiflora*. The latter infraspecies shows signs of several unique translocations and has a significantly lower percentage of transposable elements (TE), than the genome of *C. hispida* var. *hispida*. This fact may be a potential explanation for the previously observed difference in relative genome size between these two taxa (Wu, 2016). At the same time, the subgenome H^7^ of *C. sativa* and the genome of *C. hispida* var. *hispida* have a similarly large number of TEs and also share a TE location in syntenic regions almost twice as often as H^7^ and *C. hispida* var. *grandiflora* do (Martin et al., 2022).

Moreover, such evidence of genome-wide differences between *C. hispida* var. *grandiflora* and *C. hispida* var. *hispida* could be an additional issue for revision of their taxonomic status and probably the further rehabilitation of these taxa at the species rank. The taxonomy of two additional varieties of *C. hispida* (var. *lasiocarpa* and var. *stiefelhagenii*) is also discussed by Mutlu and Karakuş (2019). Acceptance of these two varieties of *C. hispida* as separate species is also under debate (Al-Shehbaz and Barriera, 2019), as well as their role in the genus evolution. Further involvement of molecular genetic analysis could help facilitate this investigation.

### 2.5 Divergence of hexaploid species was accompanied by gradually limiting genetic diversity

The vast majority of studies indicate extremely limited genetic variability in *C. sativa* accessions (Galasso et al., 2015; Singh et al., 2015; Luo et al., 2019; Blume et al., 2020; Chaudhary et al., 2020). This is well explained by the evolutionary history of *C. sativa*, as this crop has encountered at least three major bottlenecks limiting its genetic diversity. The first of such events of narrowing genetic diversity occurred during the formation of tetraploid *C. intermedia*, the second during its hybridization with *C. hispida*, which led to the emergence of hexaploid *C. microcarpa* type 1, and, finally, the third, when domesticating wild *C. microcarpa* and further breeding *C. sativa* crop. During all of these three stages, only a limited number of genotypes or/and populations have participated in allopolyploidization, crucially narrowing the diversity of descendant species. These bottlenecks are marked in the evolutionary diagram, shown in **Figure 1**.

A significant narrowing of the genetic variability of *C. sativa* was also caused by self-pollination of this species and its ancestors (except *C. hispida*). Kim et al. (2017) noted that the paucity of genetic diversity of cultivated *C. sativa* was caused not only by strong directional selection in the past (purifying selection, including wild progenitors), which may have occurred during domestication of this crop and its subsequent selection, but also by the high level of self-compatibility of *C. sativa* (and its ancestors, as we know now), combined with high selective pressure during the evolution this taxa. Most extant *C. sativa* cultivars have a very low heterozygous rate, indicating that this is due to the inbred nature of this crop, resulting from self-compatibility and common self-pollination (Manca et al., 2012; Blume et al., 2020).

Finally, the abandonment of *C. sativa* cultivation during the twentieth century (due to the predominant cultivation of rapeseed, sunflower, etc., in Europe) led to loss of the existing varietal diversity of *C. sativa* (European Commission, 2017). However, regions of previously active cultivation of *C. sativa* may have contained a fraction of varietal/landraces in the wild. For instance, in Ukraine, which was the place of widespread cultivation of *C. sativa* until the 1940s–50s (Iljinska et al., 2007; Blume et al., 2022), wild (escaped from fields) populations of this crop and of *C. alyssum* can be found (Brock et al., 2020). Previously, Ghamkhar et al. (2010) reported that the Eastern European region (in particular, Ukraine) may be a hotspot for the hexaploid *Camelina* genetic diversity. Sometimes, such wild populations of *C. sativa* can be found across the European Plain, the Caucasus, the Caspian region, and the eastern part of Central Asia (Čalasan et al., 2019).

The limited genetic variability among *C. sativa* accessions is apparently a result of the gradual narrowing of genetic diversity described above. This fact along with the limited number of accessions available in gene banks significantly limits the efficient propagation of this emerging biofuel crop. However, there are some possibilities to overcome genetic paucity of *C. sativa*, particularly introgression of genes from wild populations of the species or from its wild relatives, as well as resynthesis of this crop from its potentially ancestral species, enabled by the understanding of the genus evolution. These possible approaches will be discussed below.

## 3 Higher genetic diversity among wild *Camelina* species opens broad perspectives for interspecific hybridization

### 3.1 Diploid *Camelina* species cannot directly hybridize with allohexaploid *C. sativa*

Wild *Camelina* species can be an extremely valuable source of germplasm for overcoming the limited genetic variability of allohexaploid *C. sativa*. Although it has been described above that the limited diversity of *C. sativa* is largely due to the self-pollination nature of this plant, it can be expected that self-incompatible diploids may show much higher levels of genetic polymorphism. Galasso et al. (2015) observed no genetic variation in individual plants of a particular lineage/affinity of *C. sativa*, *C. microcarpa*, *C. alyssum*, and *C. rumelica*. In contrast, *C. hispida* (var. *grandiflora*) had the highest level of genetic polymorphism within one line, whereas *C. laxa* had a lower, but still very significant, variation among individuals within a particular line. Such difference in levels of genetic polymorphism is well consistent with the self-incompatible nature of these two species. Additionally, Chaudhary et al. (2020) noted that wild tetraploid and, especially, diploid *Camelina* species have greater genetic diversity than any of the extant hexaploids, even more than any of *C. microcarpa* cytotypes. From this perspective, *Camelina* diploids appear extremely attractive as germplasm donors for *C. sativa* improvement.

**Figure 2:**
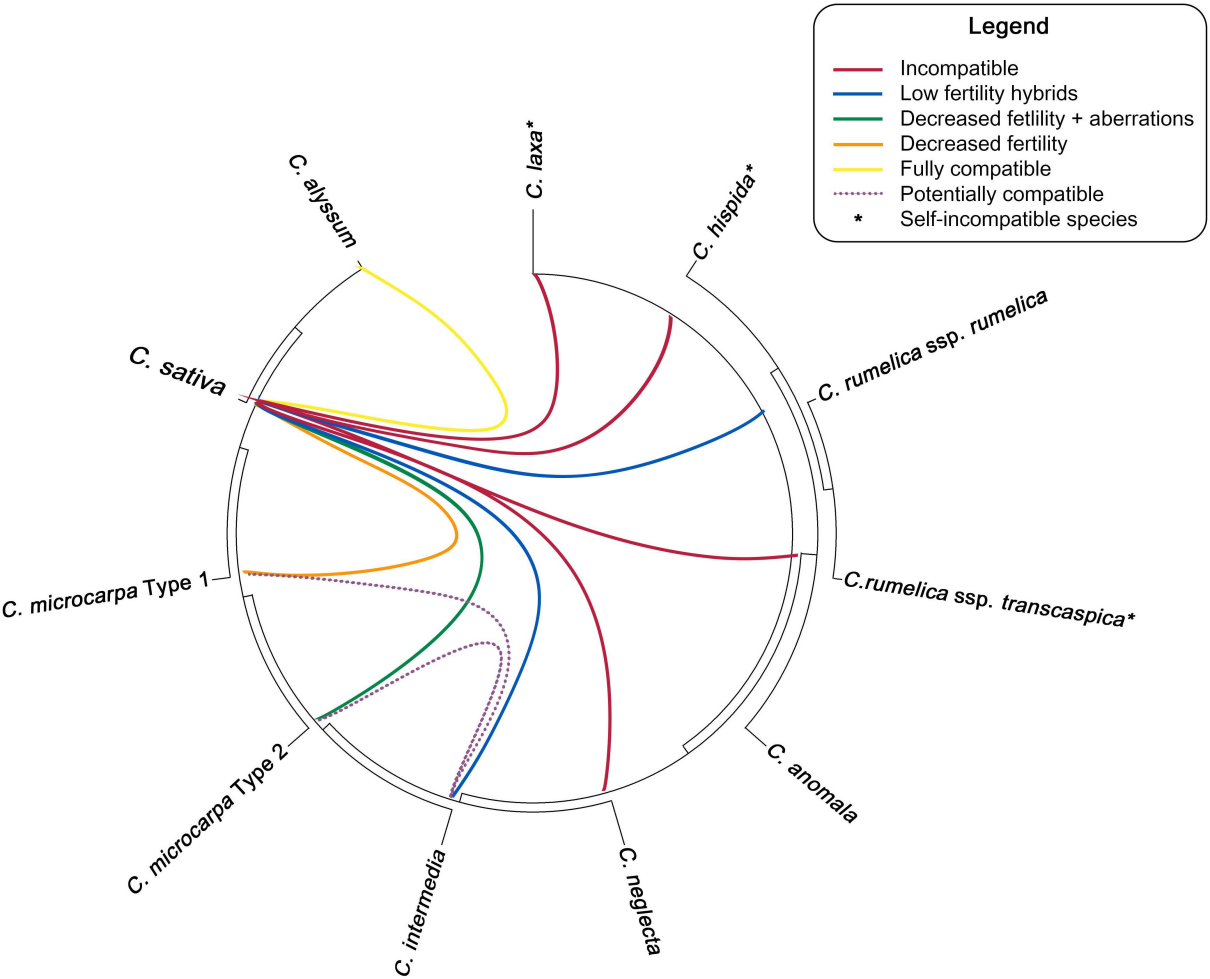
Ability of different *Camelina* species to hybridize with *C. sativa* and with each other. Phylogenetic tree topology is based the results, reported by Brock et al. (2022a).

Unfortunately, none of the diploid species can be successfully crossed directly with cultivated *C. sativa*. Martin et al. (2019) reported the inability to form hybrids between *C. neglecta* (referred to as diploid *C. microcarpa*) and *C. sativa*. Crossing of *C. laxa* and *C. hispida* with *C. sativa* resulted in production of several silicles on hybrids containing no seeds (Zhang and Auer, 2020). These results suggest that the direct hybridization between *Camelina* wild diploids and cultivated hexaploids is rather impossible. Potentially, wild-type alleles from these species could be introgressed into *C. sativa* using the tetraploid species as an intermediate acceptor for such alleles of interest. A diagram, showing the cross-compatibility of different *Camelina* species is presented in **Figure 2**.

### 3.2 Possibilities for *C. sativa* interspecific hybridization with tetraploid relatives

Using *Camelina* tetraploids for gene introgression may be a more meaningful option, as they can hybridize with *C. sativa* more successfully. The most obvious candidate for such crossing is a direct maternal progenitor of this crop—*C. intermedia* (tetraploid form of *C. microcarpa*). To date, only one study has investigated the possibility of such interspecific crossing (Martin et al., 2019). It was established that tetraploid *C. intermedia* is able to form stable hybrids with *C. sativa*, but at low rates (0.009 hybrids per flower pollinated) (**Figure 2**). Such hybrids showed reduced pollen fertility and produced limited seeds when backcrossed or selfed. Interestingly, backcrossing *C. sativa* × *intermedia* with *C. intermedia* tended to produce more seeds (Martin et al., 2019). Such low fertility of the resulting hybrids can reduce the efficiency of gene introgression into cultivated *Camelina*. In addition, it has been reported that the tetraploid form of *C. microcarpa* (≡*C. intermedia*) can coexist with hexaploid *C. microcarpa* in the wild, suggesting that there is a natural gene flow between these two taxa in nature (Martin et al., 2017). These facts suggest that potential production of *C. sativa* × *intermedia* hybrids may be somewhat successful, albeit potentially labor-intensive, in restoring normal fertility.

Although *C. rumelica* did not play an important role in the formation of *C. microcarpa*/*sativa* lineage (**Figure 1**), this tetraploid species is still of great interest for further research. This is especially true, taking into account that two *C. rumelica* infraspecies, subsp. *rumelica* and subsp. *transcaspica*, demonstrate different abilities to hybridize with *C. sativa* (**Figure 2**). Seguin-Swartz et al. (2013) reported that *C. rumelica* subsp. *rumelica* was able to hybridize with female plants of *C. sativa* and form fertile progeny. Such hybrids were reported to be highly sterile but viable and able to self-produce. At the same time, *C. rumelica* subsp. *transcaspica* failed to hybridize with *C. sativa* or the hybrid seeds formed were non-viable. In contrast, Zhang and Auer (2020) state that *C. rumelica* (most likely subsp. *rumelica*) failed to produce any hybrid seeds after crossing with *C. sativa* and in backcrosses.

A study by Zhang et al. (2022) confirmed findings of Seguin-Swartz et al. (2013). Authors of the latter study reported that hybrids of *C. rumelica* × *sativa* and *C. sativa* × *rumelica* can be obtained with almost similar success rates (1 hybrid per 217–220 ovules pollinated). It was also shown that the pollen of such hybrids had reduced viability (less than 2%), whereas the *F_2_* progeny could only be obtained by selfing, but not backcrossing, which mirrors and is consistent with the results of Seguin-Swartz et al. (2013) on *C. rumelica* subsp. *rumelica*. Zhang et al. (2022) also reported that hybrids with reduced seed productivity could inherit the winter-type life cycle from *C. rumelica* with a probability of 0.5%.

Potentially, such differences in the viability of hybrid progeny can be caused by different levels of unequal pairing of chromosomes and different chromosomal organization within subgenomes of the mentioned subspecies (e.g., the N^6^ subgenome of *C. rumelica* was completely rearranged). Nothing is known about the genome organization of *C. rumelica* subsp. *transcaspica*, so its complete inability to hybridize with *C. sativa* can hardly be explained. Additionally, nothing is known about the ability of *C. anomala* to hybridize with other species, nor about its genome and ploidy. Summarizing the given above, hybridization of *C. sativa* with tetraploid relatives may be an acceptable option but has limited success and may require further arduous breeding for fertility restoration.

### 3.3 Prospects for *C. sativa* hybridization with hexaploid *Camelina* relatives

The possibility of gene introgression from *C. microcarpa* is more often discussed, since this species is the closest relative of cultivated camelina (**Figure 1**). Crossing of *C. sativa* with its closest ancestor may allow the restoration of higher genetic diversity that was lost during domestication. Recently, *C. sativa* has been confirmed to lack a number of alleles present in *C. microcarpa* type 1 and type 2 genotypes during domestication (Chaudhary et al., 2020). However, since this wild species consists of two distinct cytotypes with the different genome organization, hybridization success may depend on the ploidy/chromosomal number of the *C. microcarpa* sample used.

The first records of interspecific hybridization of *C. microcarpa* and *C. sativa* were published by Tedin (1925), who suggested that cross was likely to be successful but that the progeny had a high level of sterility and possessed a dwarf phenotype. At that time, the chromosome count of *C. microcarpa* was obviously not taken into account. More recently, Seguin-Swartz et al. (2013) reported that hybridization success with *C. sativa* greatly depends on the particular *C. microcarpa* genotype used. The number of chromosomes of the accessions used in the study was not reported, but the authors noted that the genotypes differed in genome size. Later, Chaudhary et al. (2020) reported that *C. microcarpa* type 1 and type 2 may have differences in relative genome size.

Zhang and Auer (2020) reported that *C. sativa* and *C. microcarpa* (and vice versa) could produce hybrid progeny, suitable for backcrossing. The authors used *C. microcarpa* accessions PI633186 and PI633190, the first of which had been reported earlier to belong to type 2 (2*n* = 38), according to Chaudhary et al. (2020). Unfortunately, Zhang and Auer (2020) did not report any chromosomal rearrangements or fertility reduction in the hybrid progeny.

Recently, Tepfer et al. (2020) reported that *C. microcarpa* (accessions collected in France) were not able to form stable and fertile hybrids with *C. sativa*. *F_1_ C. microcarpa* × *C. sativa* hybrids had various meiotic abnormalities (presence of univalents, bridges, and chromosome fragments), which led to an abnormal phenotype in the hybrid progeny and led to a decrease in the fertility of interspecific hybrids. Tepfer et al. (2020) investigated the approximate ploidy of the studied samples and concluded that the number of chromosomes was “approximately” *n* = 20 (the authors did not determine the exact number of chromosomes). However, provided by Tepfer et al. (2020), microphotographs may indicate that *C. microcarpa* most likely had *n* = 19 chromosomes. Taking into account the results of Chaudhary et al. (2020), it can be assumed that the studied *C. microcarpa* of French origin, used by Tepfer et al. (2020), could belong to type 2, which experienced multiple genome restructuring events.

Recently, Chaudhary (2021) confirmed that hybridization of type 2 C*. microcarpa* with *C. sativa* (followed by *C. sativa* genome-type species, *C. alyssum*) leads to unequal recombination between chromosomes and significant chromosomal rearrangements, including the loss of certain genomic regions or resulting chromosomes. However, the example of this study shows that backcrossing such hybrids with a 40-chromosomal relative could help to restore fertility and produce stable hybrids that may be used in breeding.

On contrary, hybridization between 2*n* = 40 C*. microcarpa* type 1 and *C. sativa* can be highly successful (**Figure 2**). Martin et al. (2019) reported that such a cytotype can be crossed with *C. sativa* and viable and fertile hybrid progeny can be obtained. However, such hybrids will still show reduced pollen viability (only 17%) and decreased seed productivity. Seed size, plant height, and thousand-seed weight in hybrids were decreased and represented the average of two parental species. The possibility of the gene flow between *C. microcarpa* and *C. sativa* is supported by findings of mixed populations in the nature (Martin et al., 2017).

*C. microcarpa* type 2 was found to have the highest number of minor alleles in the first subgenome (derived from *C. neglecta*) and the highest number of unique minor alleles (over 1000), compared with C*. microcarpa* type 1 and *C. sativa*. At the same time, the third subgenome (H^7^) of *C. microcarpa* type 1 has more such minor alleles than the third subgenome (N^6^-type) of type 2 does. Some of these minor alleles may be of interest for *C. sativa* breeding, so introducing them from both types of *C. microcarpa* appears to be a promising approach to overcome the limited genetic diversity of false flax crops. Hopefully, there are a number of studies that reveal the association of agronomically important traits with certain loci in the *C. sativa* genome, which has allowed the identification of genes, involved in the shaping of the productivity of this crop (Gehringer et al., 2006; Li et al., 2021).

In addition, populations of *C. microcarpa* and wild *C. sativa* can be screened for useful allelic variants for further gene introgression. Brock et al. (2020) found that *C. microcarpa* type 2 populations of Ukrainian origin may contain more genetic diversity than type 1 representatives, collected from the Caucasus (potentially the entire range of type 1 distribution). The latest results also indicate that the Ukrainian population of *C. microcarpa* has a certain genetic heterogeneity (Sakharova et al., 2023). This is consistent with previous findings that reported high genetic diversity of hexaploid *Camelina* sp. in Eastern Europe (Ghamkhar et al., 2010). However, the cytotype of *C. microcarpa* should always be taken into account because of the potential difficulties, associated with crossing of hexaploid *Camelina* with different chromosome numbers. Currently, no tools have been proposed for efficient and high-throughput recognition of *C. microcarpa* cytotypes (e.g., DNA-barcoding tools), but some studies have been conducted to address this problem (Galasso et al., 2015; Brock et al., 2022a; Mandáková and Lysak, 2022; Sakharova et al., 2023).

Improvement of *C. sativa via* interspecific crossing may not be limited to hybridization with *C. microcarpa*, but other hexaploid species/forms should be considered (**Figure 2**). For instance, *C. alyssum* has several beneficial agronomic traits, including significantly enlarged seeds and seed pods. In addition, all available studies show that *C. alyssum* is fully interfertile with *C. sativa* (Tedin, 1922; Seguin-Swartz et al., 2013; Zhang and Auer, 2020; Chaudhary, 2021). Moreover, the intermediate phenotype of *C. alyssum* x *sativa* hybrids might not have the reduction in seed or pod size that would be expected in the case of *C. microcarpa* × *sativa* hybrids. This would significantly simplify further breeding and selection of hybrids for higher plant productivity.

Another option could be the crossing of cultivated *C. sativa* with its winter forms or isolated populations of potentially separate infrataxa—subsp. *pilosa*—as such winter plants were found to be genetically distinct from majority of existing spring varieties of *C. sativa* (Kim et al., 2017; Chaudhary et al., 2020). In this case, no difficulties are expected, associated with chromosome instability in hybrid progeny or with reduction of plant productivity. In addition, it has recently been shown that hybrids between winter and spring forms of *C. sativa* will have an intermediate phenotype with prolonged vegetation cycle (terms are intermediate between those of the parental genotypes) and often do not require vernalization (Chaudhary, 2021). Crossing genetically distant varieties or obtaining doubled haploid lines from hybrids can be an additional considerable option to increase *C. sativa* genetic diversity (Zelt and Schoen, 2016; Sadeghikian et al., 2022).

**Figure 3:**
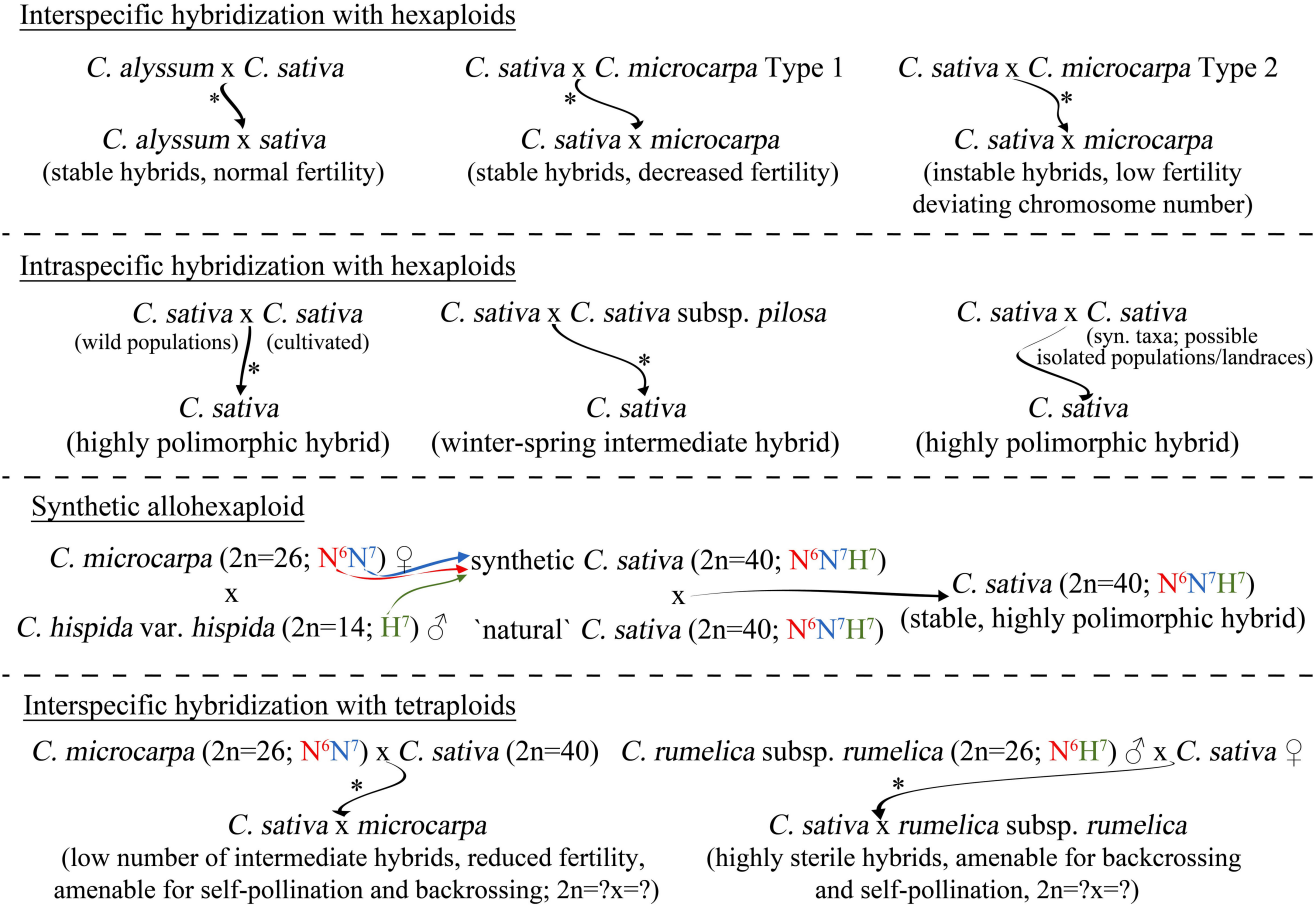
Possible approaches to overcome the genetic paucity of *C. sativa *via interspecific hybridization. Represented approaches rely on the proposed evolutionary model or on the previously reported experimental data (denoted with *).

However, the use of these approaches is limited by the number of *Camelina* sp. accessions, available in major gene banks (USDA-ARS, IPK Gatersleben, PGRC, etc.). For example, the diversity of *C. alyssum* is represented with only a few samples (1–3, depending on the gene bank), which may not be enough for effective breeding. From this point of view, the possibility to use plant germplasm (including wild populations of *C. sativa*) collected from the wild nature should be considered. Luckily, recent research on the diversity of hexaploid *Camelina* species offer information on the distribution of these species and the possible hotspot of the diversity of these species. The considerable candidates for interspecific hybridization with *C. sativa* are summarized in **Figure 3**, as well as a proposed potential approach for the resynthesis of *C. sativa* which is described in the next section.

### 3.4 Understanding of evolution enables resynthesis of hexaploid *C. sativa*

Another interesting approach that is currently being widely discussed is the resynthesis of allohexaploid *C. sativa* (Brock et al., 2019; Brock et al., 2022a; Martin et al., 2022). The proposed integrated evolutionary model of the *Camelina* genus (**Figure 1**) made it possible to clarify the most important questions of the origin of hexaploid species, in particular the origin of *C. sativa*, enabling further research toward the successful resynthesis of this complex allohexaploid from its diploid and tetraploid relatives (**Figure 3**). At the current stage, the creation of a synthetic hexaploid *Camelina* based on the existing tetraploid form of *C. intermedia* (N^6^N^7^) (Mandáková and Lysak, 2022) and diploid species *C. hispida* (H^7^) seems to be the most promising in particularly using var. *hispida*, as it was defined as the most likely exact progenitor of the third subgenome (Mándaková et al., 2019; Martin et al., 2022). There is a high probability that the use of another var. of *C. hispida* for resynthesis may lead to instability of hybrid progeny of synthetic and natural *C. sativa*. *C. hispida* var. *grandiflora* maintains much lower collinearity with the karyotype of Cs-G3, which may lead to problems similar to those observed in hybridization between *C. microcarpa* type 2 and *C. sativa*. It is also important that during such resynthesis the tetraploid *C. intermedia* should be a maternal plant to keep proper cytoplasmic inheritance, as it was during the evolution of *C. sativa* (**Figure 1**). Cytoplasmic inheritance can affect further hybrid progeny ability to self-pollinate or be compatible with the wild natural *C. sativa*, since, for example, mitochondrial genes play an extremely important role in self-pollen acceptance or rejection (García-Valencia et al., 2017).

A successful example of creating synthetic hexaploids within the *Brassica* genus by crossing tetraploid and diploid species suggests that the proposed approach for *Camelina* may also be highly realistic (Gaebelein et al., 2019). In addition, the use of synthetic *Brassica* polyploids proved to be an extremely efficient tool for rapid boost of phenotyping variation, which can significantly promote breeding (Zhang et al., 2016).

Since diploids cannot be directly crossed with hexaploid *C. sativa*, the only possible way to exploit the diversity of the highly polymorphic germplasm of these species is to use intermediate acceptor of either a resynthesized hexaploid, or naturally occurring tetraploid (Eeckhaut et al., 2006). As the *C. neglecta* cannot hybridize with *C. sativa* directly, it could be firstly crossed with tetraploid *C. microcarpa*, which can then be hybridized with hexaploid *C. sativa*. The same approach can be applied to *C. hispida* and *C. rumelica*. However, in it is currently unknown whether this approach will be successful, since no study has reported the ability of diploid and tetraploid *Camelina* species to form hybrids. Tetraploid *C. microcarpa* and *C. rumelica* subsp. *rumelica* may be of particular interest as donors of the wild germplasm diversity (**Figure 3**). The recently discovered wide range of geographical distribution of tetraploid *C. microcarpa* (≡*C. intermedia*) (Čalasan et al., 2019; Brock et al., 2022a; Mandáková and Lysak, 2022) suggests that some populations of this cytotype may be sufficiently genetically distinct to be considered as a potential source of allelic variation. The reported ability of tetraploid *C. microcarpa* to form stable hybrids with *C. sativa* (Martin et al., 2019) greatly expands the possibilities of introgression of the wild gene pool into cultivated *C. sativa*. However, such hybrids will have an intermediate phenotype between the parental species, which may result in reduced seed productivity of such hybrids.

*C. rumelica* is also able to form fertile hybrids with *C. sativa*, but less successfully: only as a paternal plant when crossed with *C. sativa*. Another problem is the high sterility of such hybrid progeny, which leads to a poor ability to produce seeds in subsequent back- or self-crossing. It is likely that separate evolutionary histories (different allopolyploidy trajectories and distinct cytoplasmic inheritance) set barriers to efficient interspecific hybridization of *C. rumelica* with *C. sativa*. However, this research direction is of considerable interest. Since *C. rumelica* plants are often found in regions with arid climate (Brock et al., 2018; Brock et al., 2020) or on the shores of seas or salt lakes (Iljinska et al., 2007), they may have a high level of tolerance to abiotic stresses, associated with increased salinity or drought. The introgression of such traits in *C. sativa* is of considerable interest, as the yields of this crop are significantly limited by water scarcity in arid regions (Estakhr and Ranjbar, 2021; Gore and Kurt, 2021; Neupane et al., 2022). However, this topic requires further research, especially regarding the incompatibility of *C. sativa* and *C. rumelica* subsp. *transcaspica*, which may be caused by genes involved in self-incompatibility reactions, as observed in other species (Morimoto et al., 2020). Crossing the two tetraploids 4x *C. microcarpa* and *C. rumelica* should also be considered, as a potential hybrid could benefit from crossing wild germplasm with *C. sativa*. However, according to the reported results of such crossing within *Brassica* genus (Katche et al., 2021), such a hybrid can potentially be tetraploid, carrying complete N^6^ genome and the N^7^-H^7^ genome association, which may cause chromosomal instability in progeny from crossing of such a hybrid with conventional *C. sativa*. This approach requires additional research but can still be viewed as an additional opportunity for improvement of genetic variation within *C. sativa*.

### 3.5 Intergeneric hybridization of *C. sativa* with other Brassicaceae

Intergeneric hybridization of *C. sativa* with other species can also be considered as a possible approach to overcome the genetic paucity. However, to date, no significant success toward the obtaining of such intergeneric hybrids has been achieved. Crossing of *C. sativa* with *Neslia paniculata*—the closest relative of *Camelina* genus, resulted in an extremely low number of *F_1_* hybrids. When *C. sativa* was a maternal plant in the crossing, *C. sativa* x *N. paniculata* hybrids showed very low fertility and were unable to form viable progeny in self- or back-crosses (Martin et al., 2020). If *N. paniculata* was a maternal plant in the cross, neoautotetraploids of *N. paniculata* were formed.

Hybridization of *C. sativa* with more distant Brassicaceae species was also unsuccessful. Several studies reported that *C. sativa* was able to hybridize with *Capsella bursa-pastoris* (Séguin-Swartz et al., 2011; CFIA, 2012; Julié-Galau et al., 2014; Martin et al., 2015; Zhang and Auer, 2020). Obtained hybrids were viable and had intermediate phenotype between parental species, especially the shape of seedpods. However, hybrid progeny was sterile and unable to produce any seeds in self- and back-crosses (Julié-Galau et al., 2014; Martin et al., 2015). A similar outcome was observed for hybrids between *C. sativa* and *Capsella rubella* (Séguin-Swartz et al., 2011; CFIA, 2012). Potentially, such hybrids could be used for further research, including breeding, but only if the fertility is restored (Martin et al., 2015). Attempts to obtain crosses between *C. sativa* and even more distant relative *A. thaliana* brought no seeds, whereas hybridization with *Cardamine hirsuta* appeared to be more successful—several hybrid seeds were produced, albeit non-viable (Julié-Galau et al., 2014).

Crossing of *C. sativa* with other Brassicaceae, like *Brassica rapa*, *Brassica nigra*, *B. napus*, *B. juncea*, and *Thlaspi arvense*, was also unsuccessful (Séguin-Swartz et al., 2011; CFIA, 2012). Only little progress was achieved in somatic hybridization of *C. sativa* with *B. napus*, which resulted in producing hybrids of intermediate phenotype, increased content of linolenic fatty acid, and an ability to backcross with *B. napus* (Jiang et al., 2009). Somatic hybridization between *C. sativa* and *Brassica carinata* or with *Brassica oleracea* was unsuccessful (Narasimhulu et al., 1994; Hansen, 1998; CFIA, 2012).

## 4 Conclusions

The presented integrated evolutionary model of the genus *Camelina* offers the most up-to-date view on a series of evolutionary events, which led to origin of the emerging oilseed crop, *C. sativa*. The exceptional role of *C. neglecta* in the genus evolution is shown, as this species contributed into the origin of all *Camelina* polyploids, whereas cultivated hexaploid species have at least two *C. neglecta*-type subgenomes and share common maternal lineage with this diploid. Understanding the evolution of *Camelina* has revealed several crucial bottlenecks of genetic diversity that have led to the current genetic paucity of *C. sativa*, which include not only polyploidy events but also domestication and relatively recent varietal diversity loss due abandonment of this crop cultivation in the previous century. In light of this problem, interspecific hybridization with crop wild relatives appears to be a significant and extremely promising approach.

The ability of *C. sativa* to hybridize with its wild relatives has been reviewed. The general conclusion is that allohexaploid *C. sativa* is poorly hybridized with diploid species and more efficiently with tetraploids or complex allopolyploids (if somatic hybridization with *B. napus* is considered). The promising approach is hybridization of *C. sativa* with its closest relative, *C. microcarpa*. However, cytotype identity of the wild relative should be considered, since *C. microcarpa* type 2 has different genome organization, which is explained by its evolutionary history. Lastly, taking into the account knowledge about *C. sativa* evolutionary origin, a pathway for the resynthesis of this allohexaploid crop has been proposed. Such synthetic *C. sativa* might be used for gene introgression from diploid *C. hispida*, which do not hybridize with *C. sativa*, or from tetraploid *C. microcarpa* (≡*C. intermedia*), hybridization with which has had so far limited success.

All these findings suggest that the greater perspective for improvement *C. sativa* genetic variability is held by approaches, based on interspecific hybridization with close relatives from *Camelina* genus and by potential resynthesis of this allohexaploid crop.

## Author contributions

RB: Conceptualization, Formal Analysis, Visualization, Writing – original draft, Writing – review & editing. RK: Conceptualization, Funding acquisition, Methodology, Writing – original draft, Writing – review & editing. LG: Conceptualization, Funding acquisition, Project administration, Supervision, Writing – review & editing. EC: Conceptualization, Funding acquisition, Writing – review & editing. YB: Conceptualization, Funding acquisition, Project administration, Supervision, Writing – original draft, Writing – review & editing.
